# Supplementary material for: Evaluation of Efflux-Mediated Resistance and Biofilm formation in Virulent Pseudomonas aeruginosa Associated with Healthcare Infections
Source: Antibiotics (Basel). 2023 Mar 22;12(3):626. doi: 10.3390/antibiotics12030626 (PMC10044907; doi:10.3390/antibiotics12030626)
Supplement: Supplementary file 1 [file antibiotics-12-00626-s001.zip › antibiotics-1797458-supplementary.pdf]

Evaluation of efflux-mediated resistance and biofilm formation in virulent *Pseudomonas aeruginosa* associated with healthcare infections

Akinduti P.A[1*], George W.O[1], Ohore H.U[1], Ariyo O.E[2], Popoola S.T[1], Adeleye A.I[3], Akinwande K.S[4], Popoola, J.O[5], Rotimi S.A[6], Olufemi F.O[7], Omonhinmin C.A[5], Olasehinde G.I[1]

Supplementary Table S1. Primers used for amplification of isolated *P. aeruginosa.*

Gene	Primer sequences (5′ - 3′)	Product size (bp)	Annealing temperatures (°C)	Reference
mexA	F-CTCGACCCGATCTACGTC<br>R-GTCTTCACCTCGACACCC	179	56	[21]
mexB	F-TGTCGAAGTTTTTCATTGATAG<br>R-AAGGTCACGGTGATGGT	244	58	[21]
oprM	F-CCATGAGCCGCCAACTGTC<br>R-CCTGGAACGCCGTCTGGAT	205	57	[21]

Supplementary Table S2. Antimicrobial susceptibility pattern of the isolated multidrug resistant *Pseudomonas aeruginosa* strains.

Infection source	Ref number	Age (years)	Sex	CAZ	CPR	AUG	AMP	GEN	CPX	OFL	NIT
Urinary tract infections	ps01	77	M	0	6	5	9	3	0	10	10
Wound	ps02	40	F	4	9	8	7	5	10	5	6
Wound	ps03	75	F	5	12	12	5	20	2	22	4
Urinary tract infections	ps04	5	M	9	10	9	11	20	11	24	5
Blood Stream Infection	ps05	10	M	10	10	5	13	20	10	22	2
Blood Stream Infection	ps06	13d	M	3	12	3	16	22	9	20	1
High vaginal infection	ps07	23	F	8	26	10	10	16	5	20	10
Urinary tract infections	ps08	25	F	4	26	0	6	20	3	20	3
Wound	ps09	60	F	11	22	5	8	22	2	24	6
Otorrhea	ps10	60	F	6	4	4	5	3	3	3	8
Otorrhea	ps11	23	M	3	28	8	9	20	6	24	15
Otorrhea	ps12	3	F	11	26	2	23	18	5	18	5
Otorrhea	ps13	3	F	12	26	3	21	20	4	18	8
Otorrhea	ps14	3	F	5	26	7	20	17	9	18	5
Otorrhea	ps15	3	F	4	16	5	26	20	5	18	6
Otorrhea	ps16	27	F	2	20	4	12	20	9	26	3
Otorrhea	ps17	28	F	1	12	9	0	14	2	18	3
Otorrhea	ps18	27	F	10	16	4	21	28	3	20	6
Wound	ps19	30	F	9	10	3	5	18	2	28	2
Otorrhea	ps20	1	F	7	8	2	3	9	7	9	4
Otorrhea	ps21	6	M	4	5	8	5	5	4	10	9
Otorrhea	ps22	18	F	4	14	8	1	18	5	20	8
Otorrhea	ps23	22	F	6	14	7	12	20	3	22	16
Wound	ps24	68	M	3	4	15	9	18	8	7	3
Wound	ps25	4	F	9	5	11	11	7	8	3	4
High vaginal infection	ps26	39	F	11	5	13	10	4	4	5	2
Urinary tract infections	ps27	32	F	2	12	6	4	28	10	22	4
